# Mechanical Interaction of the Pericardium and Cardiac Function in the Normal and Hypertensive Rat Heart

**DOI:** 10.3389/fphys.2022.878861

**Published:** 2022-05-02

**Authors:** Emilio A. Mendiola, Michael S. Sacks, Reza Avazmohammadi

**Affiliations:** ^1^ Computational Cardiovascular Bioengineering Laboratory, Department of Biomedical Engineering, Texas A&M University, College Station, TX, United States; ^2^ James T. Willerson Center for Cardiovascular Modeling and Simulation, Oden Institute for Computational Engineering and Sciences, Department of Biomedical Engineering, The University of Texas at Austin, Austin, TX, United States; ^3^ J. Mike Walker ’66 Department of Mechanical Engineering, Texas A&M University, College Station, TX, United States; ^4^ Department of Cardiovascular Sciences, Houston Methodist Academic Institute, Houston, TX, United States

**Keywords:** pericardial remodeling, pulmonary hypertension, right ventricle, cardiac mechanics, cardiac modeling

## Abstract

The pericardium is a thin connective tissue membrane that surrounds the heart and is an integral regulatory component of cardiopulmonary performance. Pathological growth and remodeling of the right ventricle (RV) stemming from structural heart diseases are thought to include a significant role of the pericardium, but its exact role remains unclear. The objective of this study was to investigate potential biomechanical adaptations of the pericardium in response to pulmonary hypertension and their effects on heart behavior. Integrated computational-experimental modeling of the heart offers a robust platform to achieve this objective. We built upon our recently developed high-fidelity finite-element models of healthy and hypertensive rodent hearts via addition of the pericardial sac. In-silico experiments were performed to investigate changes in pericardium reserve elasticity and their effects on cardiac function in hypertensive hearts. Our results suggest that contractile forces would need to increase in the RV and decrease in the left ventricle (LV) in the hypertensive heart to compensate for reductions in pericardium reserve elasticity. The discrepancies between chamber responses to pericardium addition result, in part, from differences in the impact of pericardium on the RV and LV preload. We further demonstrated the capability of our platform to predict the effect of pericardiectomy on heart function. Consistent with previous results, the effect of pericardiectomy on the chamber pressure-volume loop was the largest in the hypertensive RV. These insights are expected to motivate further computational investigations of the effect of pericardiectomy on cardiac function which remains an important factor in surgical planning of constrictive pericarditis and coronary artery bypass grafting.

## 1 Introduction

Efficient operation of the cardiopulmonary system requires precisely coordinated performance of the left (LV) and right ventricles (RV) (Lu et al., 2001). The pericardium has been shown to play a central role in maintaining proper ventricular coupling and preventing excessive ventricular distension (Glantz et al., 1978; Janicki and Weber, 1980; Santamore and Dell’Italia, 1998). Cardiac growth and remodeling (G&R) as a result of structural heart diseases (SHDs), such as pulmonary hypertension (PH) and myocardial infarction, may induce changes in the biomechanical behavior of the pericardium, potentially compromising its fundamental role and upsetting the balanced function of the pericardial-ventricular complex. PH, caused by progressive pulmonary vasoconstriction, leads to increased ventricular pressure that often transitions to progressive dilation of the RV. Analysis of changes in the function of the pericardium as a consequence of G&R induced by PH could provide insight into the impact of the pericardium on disease progression and cardiac function. Despite the current understanding of the pericardium as an integral component of cardiopulmonary performance, the effect of the pericardium on ventricular interaction and organ-level cardiac function in the presence of SHDs, and in particular PH, remains understudied.

Under normal conditions, the pericardium, through its close anatomic relationship with the ventricles, acts as a regulator of chamber distension and ventricular interactions, thus impacting overall cardiac performance (Janicki, 1990). The pericardium affects both diastolic and systolic function of the heart by regulating the amount of preload and ventricular interdependence (Glantz et al., 1978). Although the pericardium is known to act as a mechanical constraint against pathological variations in heart size under *acute* pressure overload (Belenkie et al., 2004), previous experimental studies suggest that the pericardium may lose its functionality in regulating cardiac function once the heart is subjected to *chronic* pressure overload (Prindle et al., 1973; Blanchard and Dittrich, 1992). This finding becomes very important in PH as the RV systolic pressure and RV volume can significantly increase and persist in PH, thus prompting the question of whether the pericardium stretching imposed by RV dilation compromises pericardial-ventricular interaction in post-PH hearts. However, the majority of studies on the function of the pericardium make use of an isolated *ex-vivo* heart, providing limited insights in studying the effect of pericardium *in vivo*. Recent advancements in integrated computational-experimental modeling offers the possibility of estimating such an effect *in silico*. Moreover, *in vivo* ventricular hemodynamic measurements are expected to be different under closed- and open-chest conditions especially noting that the role of pericardium is subsided in the latter. While previous computational studies investigating the effect of the pericardium have been conducted (Fritz et al., 2013; Pfaller et al., 2019; Strocchi et al., 2020), none have combined a volumetric representation of the pericardium with subject-specific imaging and experimental data (pressure measurements and fiber distributions). In-silico cardiac simulations can provide insight into these differences, that remain poorly understood, by studying the effect of the pericardium on cardiac function.

We recently developed a biomechanical biventricular finite-element (FE) rat heart model of cardiac contraction throughout the cardiac cycle of both normal and post-PH states. (Avazmohammadi et al., 2019a). In this work, we extended the heart models by the inclusion of the fibrous pericardium to investigate alterations in pericardial-ventricular interactions in the normal and hypertensive heart. Hereafter, we refer to the fibrous pericardium as the pericardium and to the visceral pericardium as the epicardium or heart surface. The extended models were used to test the hypothesis that the gradual dilatation of the RV throughout the development of PH, and concurrent stretching of the pericardium, compromises the integral regulatory role of the pericardium by accentuating its restrictive characteristics. We further used our model to predict the effect of pericardiectomy on normal and hypertensive hearts.

## 2 Materials and Methods

Image-based FE biventricular heart models from control (normal) and post-PH (4 weeks) rat hearts were developed (Avazmohammadi et al., 2019a). Briefly, PH was induced in rats by subcutaneous injection of a moderate dose of monocrotaline (Hessel et al., 2006); the control group received a similar injection of phosphate buffered saline (Avazmohammadi et al., 2019a). Terminal hemodynamic measurements were collected at 4 weeks post-injection with the pericardium removed. High-resolution magnetic resonance imaging (MRI) and diffusion tensor-MRI (DT-MRI) scans were performed on the prepared hearts. We used an inverse modeling approach, with experimental P-V data as input, to estimate the *in-vivo* passive and active properties of RV and LV myocardium at normal and hypertensive states. The estimated material parameters produced model behavior that closely matched the experimental data. Additional details regarding the animal model, measurements, and inverse model can be found in our previous work (Avazmohammadi et al., 2019a). In this work, these models were extended by the addition of the parietal pericardial sac and used to investigate the influence of the pericardium on myocardium kinematics and contractility in post-PH hearts.

### 2.1 Myocardium Model

Myocardium was modeled as a hyperelastic transversely isotropic material characterized by one fiber direction. The passive behavior of the myocardium was characterized by the following Fung-type strain energy function (Guccione et al., 1991)

$$W\left(E\right)=\frac{c}{2}\;\left\{e^{\alpha \left[B_{1}\;E_{11}^{2}+B_{2}\;\left(E_{22}^{2}+E_{33}^{2}+2E_{23}^{2}\right)+B_{3}\;\left(E_{12}^{2}+E_{13}^{2}\right)\right]}-1\right\},$$ (1)

where **E** is the Green-Lagrange strain tensor, and *c*, *α*, *B*_1_, *B*_2_, and *B*_3_ are material constants. The strain components refer to the local preferred material directions with the axis “1” denoting the fiber direction. The active behavior was characterized by the following stress-like function of the fiber strain (*E*_*f*_ = **e**_1_ ⋅**Ee**_1_)

$$T_{a}\left(E_{f}\right)=T_{{\text{Ca}}^{+2}}\;\left[1+\beta \;\left(\sqrt{2E_{f}+1}-1\right)\right],$$ (2)

where $T_{{\text{Ca}}^{+2}}$ is the active force parameter and *β* is a factor representing Frank-Starling effect of changing preload. The myocardial passive and active parameters in normal and post-PH heart models, without pericardium, were determined previously through an inverse modeling approach to match the organ-level pressure-volume (P-V) measurements (Avazmohammadi et al., 2019a). As the passive myocardial behavior is determined by intrinsic myocardial properties, the passive material parameters characterizing myocardial behavior were not altered after addition of the pericardium to the heart models. Further details regarding the implementation of the active and passive constitutive model can be found in the supplementary materials of Avazmohammadi et al. (2019a).

### 2.2 Integrated Rat Heart and Fibrous Pericardium Models

**FIGURE 1:**
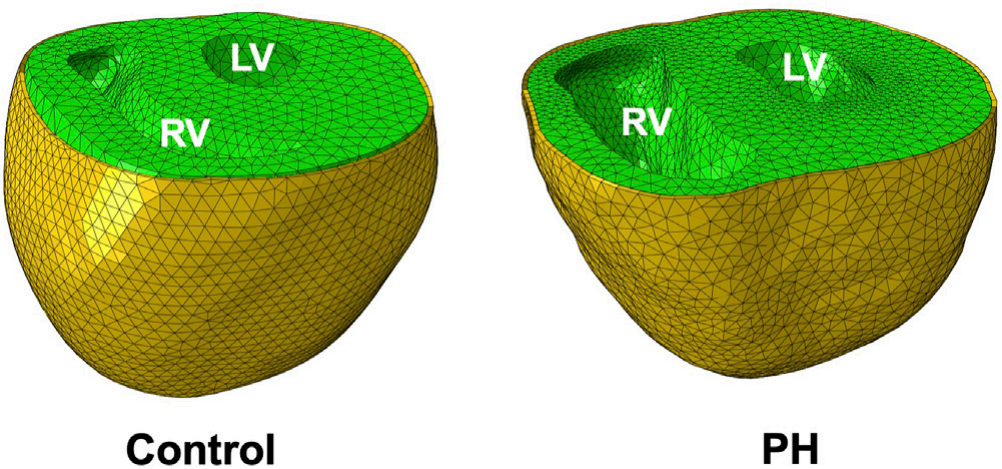
Finite-element models of control and PH rat hearts (green) and pericardium (yellow). The PH RV dilates considerably in response to pathological pressure overload.

The rat heart models were constructed from anatomical (MRI) and structural (DT-MRI) imaging data. An animal-specific pericardium was incorporated by the addition of a FE shell adjacent to the epicardium (Figure 1) made up of linear triangular shell elements. The shell was created as a distinct structure with a thickness of 10 *μm*, which is consistent with experimentally determined measurements of rodent pericardium thickness (Wang et al., 2020). Nodes on the base of the heart and on the top surface of the pericardium were constrained to the base plane. Interaction between the epicardium and pericardium was modeled by a linear penalty method (Fiacco and McCormick, 1969) as a frictionless sliding contact, accounting for the lubricating effect of intrapericardial fluid. A “no separation” condition was enforced, ensuring constant contact between the pericardium and the epicardium throughout the cardiac cycle. The same constitutive model for the passive behavior of the myocardium (Eq. 1) was used for the pericardium.

### 2.3 Estimation of Pericardial Parameters

#### 2.3.1 Control Heart

**FIGURE 2:**
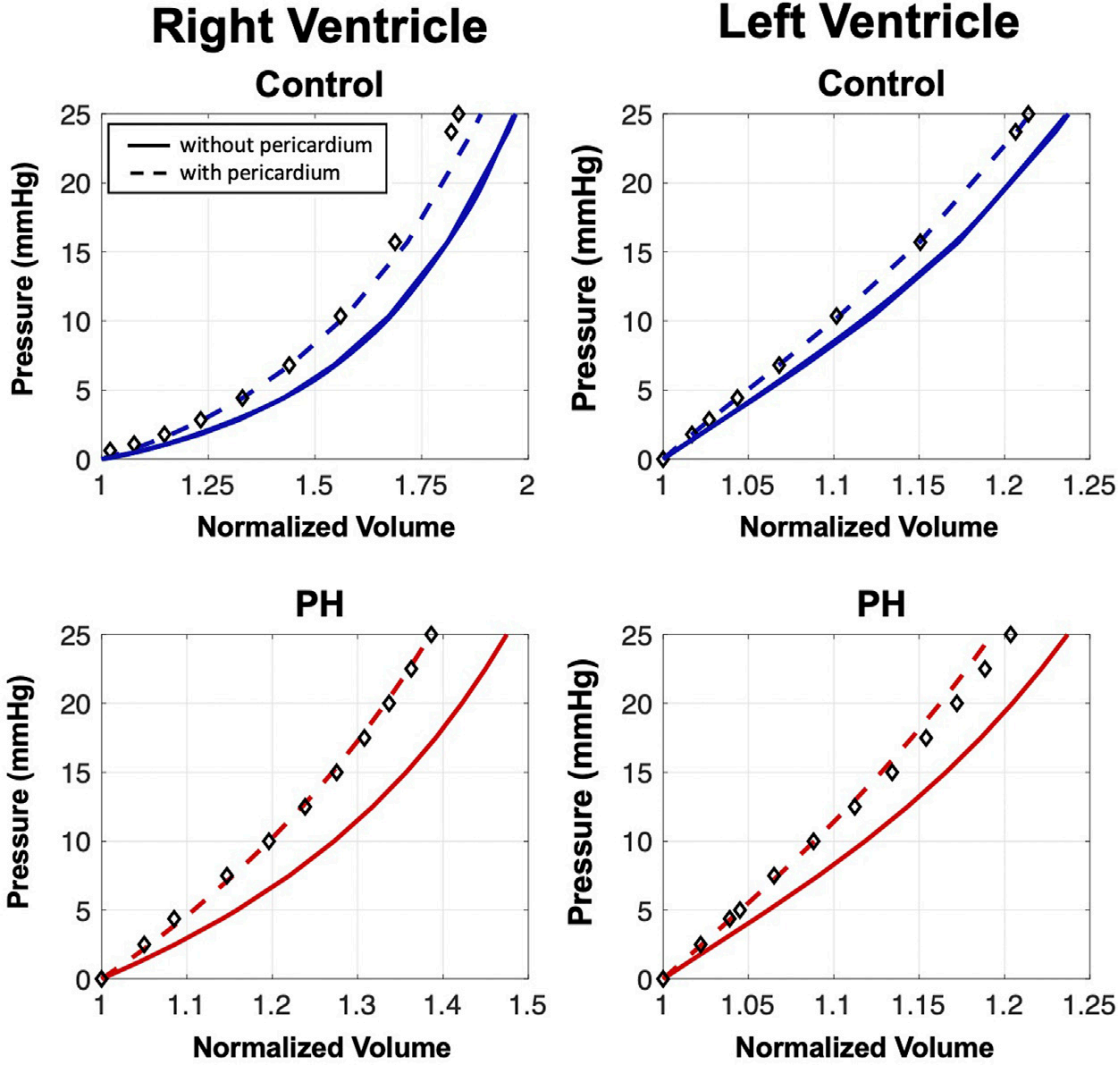
End-diastolic pressure-volume relationships (EDPVRs) resulting from simultaneous passive ventricular inflation. Curves denote heart model predictions and markers indicate the adjusted experimental fitting data for the model with pericardium (Janicki and Weber, 1980). Addition of the pericardium results in a leftward shift of the EDPVR. The pericardium has a larger influence on the passive filling characteristics of the PH heart.

The pericardial stiffness parameters, *α*_*P*_ and *c*_*P*_, in the normal heart were estimated by matching the end-diastolic pressure volume relationship (EDPVR) of hearts with pericardium. As the experimental P-V data used to estimate material properties for the previously developed heart models (Avazmohammadi et al., 2019a) was collected without the pericardium intact, the percent volume change calculated from measurements determined experimentally by Janicki and Weber (1980) were used to estimate an EDPVR for our heart models including the pericardium. An inverse modeling approach, consisting of simultaneous loading of the ventricles to 25 mmHg and calculation of the EDPVR, was used to identify the material parameters that ensured the EDPVR exhibited the desired volumetric changes (Figure 2). The material parameters were chosen such that the difference in volumes at the end-diastolic (ED) pressure-volume point from models with and without pericardium matched the percent volume change that was calculated from the experimental data.

We subsequently used the updated ED volume from the diastolic behavior of the heart model with pericardium to scale the entire P-V loop. To this end, we made use of earlier clinical and animal observations that the stroke volume (SV) remains nearly unaffected following pericardiectomy in hearts with non-infected pericardium (Berglund et al., 1955; Mangano, 1980). Having generated an estimated “experimental” P-V loop of the heart with intact pericardium, we performed inverse modeling to estimate $T_{{\text{Ca}}^{2+}}$ , characterizing the active behavior of the normal heart with pericardium (Avazmohammadi et al., 2019a).

#### 2.3.2 Post-PH Heart

To the best of our knowledge, there have been no measurements of pre- and post-pericardiectomy diastolic P-V measurements in the hypertensive heart. As such, we made an approximation as to estimating the material properties of the pericardium in the PH heart, summarized by the following remarks.

• Although the pericardium can experience fibrosis due to infection, such as constrictive pericarditis (Leak et al., 1987), there is no reported evidence that fibrosis is present in the chronically stressed pericardium (Prindle et al., 1973). Thus, the content and stiffness of collagen fibers, as the main structural components of pericardium, remain the same from normal to PH states.
• However, the pericardium must stretch to accommodate significant ventricular dilation in hypertensive hearts (as seen in the PH heart in our study.) As a connective tissue, this stretch alters the *toe region* of the tissue (the region of the mechanical response where collagen fibers are fully recruited) leading to an increase in apparent stiffness of pericardium.
• We estimated this increase by calculating the amount of stretch in pericardium induced by RV dilation in the PH heart and the relative stiffening due to the shortening of toe region. We used existing biaxial mechanical data available for bovine and canine pericardium (Choi and Vito, 1990; Sacks and Chuong, 1998) and estimated the relative changes in pericardial stiffness due to reductions in toe region length.
• The material parameters of the pericardium were prescribed to the PH heart model such that the stress-strain response was scaled appropriately.

Similar to the case of the normal heart, a P-V loop for the PH heart with intact pericardium was estimated using the changes in the diastolic behavior and maintaining the SV, and inverse modeling was performed to estimate $T_{{\text{Ca}}^{2+}}$ for the PH heart with pericardium.

## 3 Results

### 3.1 Reduction in Ventricular Distensibility

A 35% increase in the area of pericardium from the control to the PH heart decreased the toe region by 25% in extensional strain and stiffened the pericardium by 48% (Table 1). Removal of the pericardium resulted in a rightward shift of the EDPVR curve (Figure 2), indicating the pericardium is indeed acting as a constraint to ventricle distension. Although the removal of the pericardium resulted in a 5% and 2% increase in the final RV and LV volumes in the control heart, respectively, the presence of the pericardium had a greater influence on PH diastolic volumes, particularly in the RV. RV and LV volumes increased by 8% and 3%, respectively, after pericardium removal in the PH heart model. Changes in the EDPVR in both heart models after the addition of the pericardium was qualitatively consistent with the trends seen in previous experimental studies (Janicki and Weber, 1980; Santamore and Dell’Italia, 1998).

**TABLE 1:** Estimated passive properties of the RV, LV, and pericardium for the control and PH rat heart models.

[1.5 pt]	RV			LV		Pericardium	
		*α*_*RV*_	*c*_*RV*_ (kPa)	*α*_*LV*_	*c*_*LV*_ (kPa)	*α*_*P*_	*c*_*P*_ (kPa)
Control		2.07	0.43	2.07	0.52	1.5	21.13
PH		2.26	1.35	2.26	0.61	1.5	31.26

### 3.2 Contractile Adaptation and Organ-Level Performance

**FIGURE 3:**
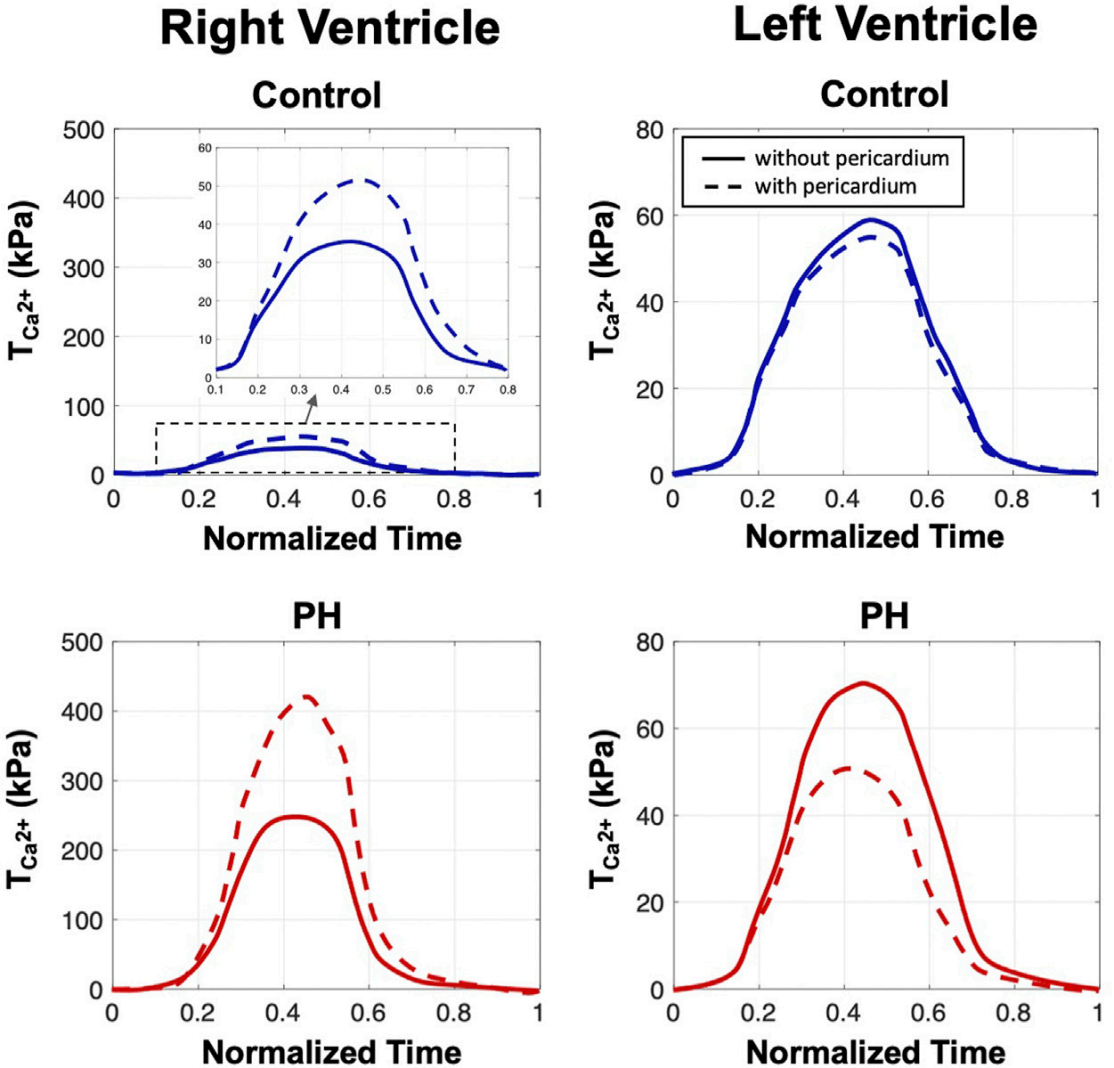
The estimated values of the active parameter $T_{Ca^{2+}}$ as a function of time for the control and PH hearts with and without pericardium. Curves estimated from the heart model with and without pericardium are denoted by w/P and wo/P, respectively.

Addition of the pericardium, while maintaining the stroke volume, caused a ∼ 50% increase in the peak value of $T_{{\text{Ca}}^{2+}}$ in the RV region of the control heart while it slightly reduced the corresponding value (7%) in the LV (Figure 3). Similar trends to that of the control heart were observed in the post-PH heart model following the addition of the pericardium, although a larger relative increase in the peak contractility were predicted. A ∼ 64% increase in RV $T_{{\text{Ca}}^{2+}}$ and a ∼29% decrease in the LV was predicted by the PH heart model.

**FIGURE 4:**
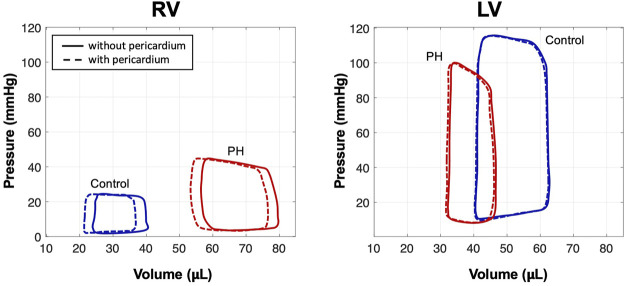
Pressure-volume loops from simulations of control and PH hearts with and without pericardium. Simulations predicted a notable increase in EDV in the RV after removal of the pericardium; EDV in the LV remained nearly constant.

Removal of the pericardium resulted in a notable increase in end-diastolic volume (EDV) in the RV in both control and post-PH cases (Figure 4). Predicted right ventricle ejection fraction (RVEF) was decreased by ∼8% and ∼4% in the control heart and PH hearts, respectively, after pericardium removal. In contrast, the predicted effect of pericardium removal was minimal on the LV EDV and LV ejection fraction (Figure 4).

### 3.3 Predicted Cardiac Kinematics

**FIGURE 5:**
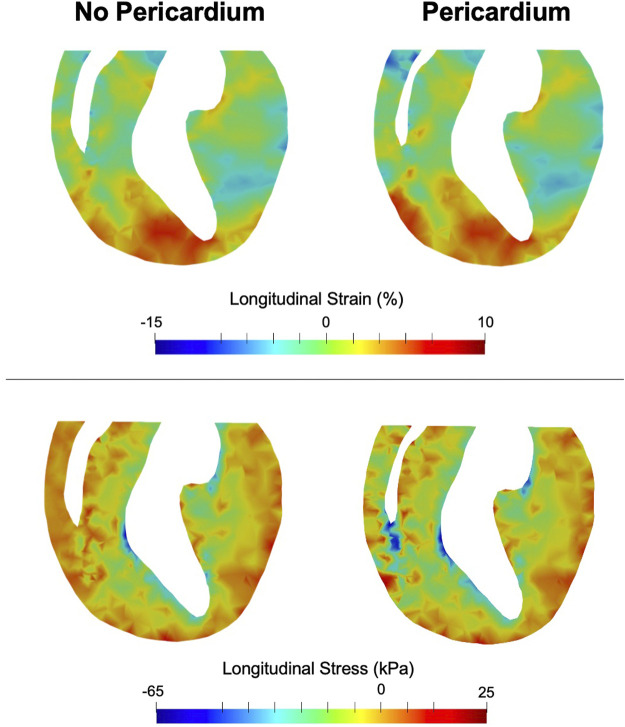
End-systolic longitudinal strain (top) and stress (bottom) in a long-axis cross section of the heart. Greater effect of the pericardium on stress can be seen in the RV rather than the LV.

Removal of the pericardium reduced longitudinal movement in both control and PH hearts. While little change was observed in the strain distributions between hearts with and without pericardium, a notable increase in RV stress was predicted in the heart models with pericardium in both control and post-PH hearts (Figure 5). No significant change was noted in LV stress.

## 4 Discussion

In this work, we have presented the first computational investigation of pericardial adaptation in the hypertensive heart. The pericardium has an innate influence on cardiac function, which could be upset by G&R events stemming from structural heart diseases. The objective of this study was to determine how the adaptation of the pericardium in the hypertensive heart impacts passive and active cardiac behaviors.

### 4.1 Adaptation of the Role of the Pericardium in the Hypertensive Heart

The mechanical restrictive effect of the pericardium on the diastolic behavior of the normal heart was consistent with the findings of pericardiectomy experiments in previous large animal studies (Janicki and Weber, 1980; Janicki, 1990). A more pronounced effect of constriction, noted in the PH heart, was likely due to significant stretching of pericardium following the RV dilation such that the pericardial “*reserve*” elasticity is minimized leading to substantial increase in the compressive pressure exerted by pericardium to the ventricles (Richardson et al., 2015). In particular, the RV in the PH heart experiences further reductions in diastolic filling indicating a *chamber-specific* compromise in the reserve elasticity of the pericardium.

As the pericardium restricts the diastolic filling (preload), and in turn limits the extent to which the Frank-Starling mechanism can be used by the heart, one could predict that the removal of the pericardium would induce a decrease in myocardial contractility necessary to maintain the SV for both ventricles. However, a rather counter-intuitive finding from our inverse model predictions was that while the RV contractility notably decreased, the LV contractility was slightly increased following the removal of the pericardium. This finding highlights the role of the pericardium in enhancing the “cooperative” RV-LV coupling as well as disparity in the changes of the RV and LV preload following the removal of pericardium. In the PH heart, removal of the pericardium induced, qualitatively similar, but quantitatively more notable, changes in the systolic behavior of the RV and LV when compared to those in the control heart. In essence, the pronounced changes in systolic function suggest that the Frank-Starling relationship is significantly altered due to excessive dilation of the RV in the PH heart such that the removal of the pericardium could significantly undermine the RV systolic function. These changes in predicted active stress are consistent with the results of Pfaller et al. (2019) who, through a computational parametric study, showed increased pericardium stiffness necessitates an increase in active stress to maintain normal cardiac output.

While this study focused on changes in cardiac behavior due to the removal of the pericardium and the potential loss of reserve elasticity in the PH case, potential alterations in the structure and content of the pericardium, manifesting as altered mechanical properties, could indeed be a mechanism by which systolic function is perturbed. Although previous studies have indicated fibrosis is not present in the *chronically* stressed pericardium (Prindle et al., 1973), it is possible that mild fibrosis and changes in the effective collagen fiber angle, as a result of geometrical changes brought on by RV dilation, contribute to impaired systolic function in addition to a reduction in reserve elasticity in the PH case. While the estimated P-V loops used to predict changes in contractility maintained a constant SV, as suggested by Mangano (1980) and Mangano et al. (1985), similar experiments were conducted with P-V loops that maintained constant EF (Berglund et al., 1955; Motoki et al., 2013; Lin et al., 2016) and results were qualitatively similar.

### 4.2 Cardiac Performance Following Pericardium Removal

The capability to perform a simulated pericardium removal was a key delivery of our integrated modeling platform. Our modeling approach, able to take into account changes in preload following pericardiectomy using subject-specific experimental data, could prove useful in predicting changes in cardiac function post-surgical intervention. A marked increase in RVEF was noted in the control heart while a less significant increase in EF was seen in the PH heart post-pericardiectomy despite a larger increase in end-diastole volume in the post-PH heart. These observations corroborate the pericardium’s enhanced restrictive effect on diastolic filling negatively impacting RVEF in the post-PH heart. At the same time, our results suggest that RV dilation and increased RV stiffness in the post-PH heart curbs the effect of pericardiectomy on enhancing RV contractility and undermines its role in maintaining ventricular interdependence.

Results collected from this computational investigation suggest that the pericardium is subjected to adaptations in the hypertensive heart that could be detrimental to cardiopulmonary performance. As observations suggest that removal of the pericardium has a notable impact on cardiac performance, pre-surgical planning in patients with structural heart disease should include a careful consideration of the effects of pericardiectomy on cardiac performance. Studies on the effect of pericardiectomy in constrictive pericarditis, which also causes diastolic dysfunction noted in this work, indicate removal of the pericardium is associated with increased risk of mortality (Bicer et al., 2015). As our modeling efforts noted large changes in contractility and stress state of the RV in PH without pericardium (Figures 3, 5), it is possible removal of the pericardium in PH may initiate detrimental remodeling events in the myocardium (Avazmohammadi et al., 2017; Avazmohammadi et al., 2019b), leading to worse outcomes as has been seen in studies of constrictive pericarditis. In addition, the pericardium plays a major role in regulating ventricular interdependence and coupling (Glantz et al., 1978; Janicki, 1990; Santamore and Dell’Italia, 1998), and results of this study warrant further investigation into alterations in ventricular interactions due to pericardial adaptation in the diseased heart. Further development of patient-specific models could ultimately lead to improved therapies and outcomes in patients with structural heart diseases.

### 4.3 Pericardial Influence on Kinematics and Stress

Given preserved SV, we expected chamber kinematics to remain similar after the removal of the pericardium (Figure 5). However, reduced pre-load in the RV with the inclusion of pericardium required a greater amount of active stress in the RV to maintain SV (Figure 5). Given that the formulation of the active model is phenomenological, the mechanisms that make up the predicted changes in active stress remain to be identified. It is possible the change in contractility is accomplished via acute stretch-dependent mechanisms, such as altered rate release of calcium from the sarcoplasmic recticulum (Gamble et al., 1992), or by longer term mechanisms, such as altered titin regulation (Herzog, 2018). When comparing changes between models with and without pericardium, the larger degree of alteration of stress in the RV, compared to the LV, suggest loss of reserve elasticity and potential remodeling of the pericardium has a greater impact on RV function, consistent with our predicted results regarding diastolic behavior (Figure 2), contractility (Figure 3), and organ-level function (Figure 4). Greater vulnerability of the RV to mechanical constraint is likely due to RV morphology and architecture, in particular RV free wall thinness.

### 4.4 Limitations

Stretch-induced stiffening of the pericardium imposed by geometric remodeling of the ventricles was assumed to be the only remodeling mechanism of the pericardium in the hypertensive heart. Additional intrinsic remodeling of the pericardium due to chronic ventricular pressure overload remains understudied and was not considered here. The fiber orientation distribution of the pericardium was taken to be the same as that of respective epicardial region in each heart. The pericardial-myocardial fiber orientation relationship and its effect on cardiac function remains to be studied, although pilot simulations suggest this effect is considered to be secondary to the effect of pericardial passive properties on cardiac function. Lastly, the changes in $T_{{\text{Ca}}^{2+}}$ in the RV and LV upon the addition of the pericardium may be quantitatively affected by the simplifying assumptions in the model, such as myocardial incompressibility and the constant effect of length-dependency on contractile force (constant factor *β* in the active stress model), although the predicted changes are expected to be qualitatively accurate.

## 5 Concluding Remarks

As existing evidences indicate that the pericardium contributes substantially to the intracavity pressures of both ventricles (Boltwood et al., 1986; Tyberg et al., 1986), understanding the change in pericardial function in response to structural heart diseases could aid in the clinical management of patients undergoing cardiac surgery and those with structural heart diseases. Further advancements of the platform presented in this work offer a pre-clinical tool that could be used to predict changes in organ-level function post-pericardiectomy.

## Data Availability

The raw data supporting the conclusion of this article will be made available by the authors, without undue reservation.
